# Inter-technique agreement of left atrial and ventricular deformation analysis: a comparison between transthoracic echocardiography and cardiovascular magnetic resonance imaging

**DOI:** 10.1186/s44156-025-00090-3

**Published:** 2025-10-06

**Authors:** Aseel Alfuhied, Jian L. Yeo, Gaurav S. Gulsin, Abhishek Dattani, Kelly Parke, Christopher D. Steadman, Manjit Sian, Anna-Marie Marsh, Gerry P. McCann, Anvesha Singh

**Affiliations:** 1 Department of Cardiovascular Sciences, University of Leicester and Cardiovascular Theme, National Institute for Health Research (NIHR) Leicester Biomedical Research Centre, Glenfield Hospital, Leicester, UK; 2 Department of Cardiovascular Technology-Echocardiography, College of Applied Medical Sciences, King Saud bin Abdulaziz University for Health Sciences, Riyadh, Kingdom of Saudi Arabia; 3 Department of Cardiology, University Hospitals Dorset, Poole, UK

**Keywords:** Transthoracic echocardiography, Cardiovascular magnetic resonance, Strain, Strain rate, Aortic stenosis, Type 2 diabetes, Left atrium, Left ventricle

## Abstract

**Background:**

Myocardial strain measurements are increasingly used in research and clinical practice. However, there are limited data on inter-modality agreement and reproducibility. We aimed to investigate the inter-technique agreement of transthoracic echocardiography (TTE) and cardiovascular magnetic resonance (CMR) imaging derived left atrial (LA) and left ventricular (LV) deformation parameters.

**Methods:**

Subjects with or without cardiovascular disease were prospectively recruited and had TTE and CMR on the same day. Ten subjects with type 2 diabetes (T2D) had both scans repeated within two weeks for test-retest reproducibility assessment. Myocardial deformation analyses were undertaken including LA strain (LAS) corresponding to LA reservoir, conduit and booster pump phases, LV global longitudinal strain (GLS) and peak early/late diastolic strain rate (PE/PLDSR) and LV mid-circumferential strain (Mid-CS) and strain rates.

**Results:**

222 participants (T2D (*n* = 87); severe aortic stenosis (*n* = 78) and healthy volunteers (*n* = 57)) were included. There were no significant differences between TTE and CMR measured LAS parameters, with moderate agreement between imaging modalities (ICC = 0.55–0.69). LV parameters were significantly higher on CMR except for Mid-CS which was higher on TTE (-19.3 ± 3.19 vs. -23.0 ± 4.37; *p* < 0.001). Inter-technique agreement was poor for all LV deformation parameters, except PLDSR with modest agreement (ICC = 0.52–0.66). CMR test-retest reproducibility was good to excellent for LAS and LV strain rate parameters (ICC = 0.73–0.90). TTE test-retest reproducibility was good for conduit LAS and LV_PEDSR (ICC = 0.80).

**Conclusion:**

There is modest agreement between TTE and CMR for LAS and poor agreement for LV strain assessment, suggesting that these techniques cannot be used inter-changeably. In a small subset of participants CMR test-retest reproducibility was overall better than TTE.

**Supplementary Information:**

The online version contains supplementary material available at 10.1186/s44156-025-00090-3.

## Background

Myocardial strain reflects the percentage change in the length of a myocardial segment throughout the cardiac cycle, whist the rate of this change is the strain rate (SR) [1]. These parameters provide a direct measure of myocardial contractility and relaxation, and are sensitive enough to allow early detection of subclinical cardiac dysfunction [2]. Strain and SR can be assessed via dedicated post-processing software (speckle tracking echocardiography (STE) or feature tracking (FT) cardiac magnetic resonance (CMR) imaging).

Strain assessment by FT-CMR has several advantages over STE such as improved spatial resolution, high signal and contrast ratio (between blood pool and myocardium), enabling optimal endocardial border tracking. Furthermore, with adequate planning, there is less propensity for foreshortened images than using transthoracic echocardiography (TTE). On the other hand, TTE has higher temporal resolution than CMR, which is essential for accurately tracking the speckles/features throughout the imaging frames [3].

Whilst left ventricular (LV) global longitudinal strain (GLS) and left atrial strain during reservoir (LAS_r) are advocated in several cardiology guidelines [4–6], both have arbitrary cut-offs based on STE measurements, despite the limitations of TTE. Good inter-technique agreement would allow the interchangeable use of imaging techniques (TTE and CMR), whilst good test-retest reproducibility is vital when monitoring treatment effects or disease progression in longitudinal studies. Although some studies have compared FT-CMR and STE for LV strain, no study has investigated inter-technique agreement for LV diastolic strain rate (LVDSR) and LAS.

In the current study, we aimed to investigate the inter-technique agreement and test-retest reproducibility between TTE- and CMR-derived LA and LV myocardial deformation parameters in a range of participants.

## Methods

### Study population

Participants were prospectively recruited for ethically approved studies at a single tertiary cardiac centre, and had TTE and CMR on the same study day. This included those with: severe aortic stenosis (AS) (aortic valve area (AVA) < 1cm^2^, mean pressure gradient (MPG) > 40mmHg or peak aortic velocity (AV-Vmax) > 4 m/s), T2D (glycated haemoglobin level ≥ 6.5%) or healthy volunteers without known cardiovascular disease (CVD). Exclusion criteria for the AS cohort were history of previous coronary artery bypass graft or valve surgery, moderate or severe disease affecting other valves and absolute contraindications to CMR. For people with T2D without CVD, the inclusion and exclusion criteria have been previously published [7, 8]. Healthy volunteers were free of cardiac disease, obesity (BMI > 30) and uncontrolled hypertension. All studies were approved by the UK National Research Ethics Service (19/EM/0032, 15/WM/0222, 17/WM/0192 and 08/H0402/6). Participants provided written informed consent prior to any testing.

### Study investigations

All subjects underwent TTE and CMR scans on the same day and within a two-hour window.

### TTE image acquisition

TTE was performed using an iE33 system with an S5-1 transducer (Philips 81 Medical Systems) or Vivid 7(GE Healthcare) by accredited cardiac physiologists. All scans were conducted according to the British Society of Echocardiography guidelines [9] and included focused images of the LA at apical 2- and 4-chamber, LV 2-, 3- and 4-chamber views and LV mid-short axis at the level of papillary muscles. All views were recorded with 40 to 80 Hz frame rate [10].

### CMR image acquisition

CMR scanning was conducted on a 3T Siemens scanner (Skyra, Erlangen, Germany) or 1.5T Siemens scanners (Avanto or Aera) with an 18-channel phased array coil. The scans included long-axis (2-, 3- and 4-chamber) cine images and short-axis LV cine stacks, using a steady-state free precession sequence (typical parameters: slice thickness of 8 mm, matrix 204 × 256, field of view variable 300–360 × 360– 420, temporal resolution 48ms at 3T and 45ms at 1.5T, echo time 1.5ms at 3T and 1.2ms at 1.5T, repetition time 3.44ms at 3T and 2.8ms at 1.5T). All acquisitions were reconstructed to 30 phases. CMR cine images were acquired at end-expiratory breath-hold, using retrospective ECG-gating. Long-axis cine images were acquired before contrast administration in all subjects.

### Test-retest reproducibility

Ten participants with T2D were invited for a repeat CMR and TTE within 14 days, with identical scanning protocols [8]. Both sets of scans were performed at approximately the same time of the day to avoid potential diurnal hemodynamic changes. Both CMR scans were conducted using 3-Tesla Siemens scanner (Skyra, Erlangen, Germany), whilst iE33 system (Philips 81 Medical Systems) was used for TTE.

### Image analysis

Image analysis was conducted by a single observer (AA) blinded to the participant details. All TTE and CMR images were assessed for image quality before analysis using the following scoring system: 0 = poor (not analysable), 1 = fair (artefact present but images still analysable), 2 = good (artefact present but not in the region of interest), 3 = excellent (no artefacts). CMR and TTE analyses were done independently of each other to minimise bias.

LA and LV volumetric quantification was conducted using CMR. LA volumes (LAVmax and LAVmin) were quantified using the biplane area length method on 2 and 4-chamber cine images, and LAEF was calculated, using QMass v8.1 (Medis Suite v3.1 Medical imaging systems). LA appendage and pulmonary veins were excluded from LA volumetric measurements. LV volumes were quantified from the short axis stack using Circle software (cvi42, 5.10, Circle Cardiovascular Imaging, Calgary, Canada). The endo- and epicardial borders at end-diastole and the endocardial border at end-systole were traced using a semi-automated technique, with manual adjustment as required. LVEF and mass were then calculated automatically by the software.

**Fig. 1:**
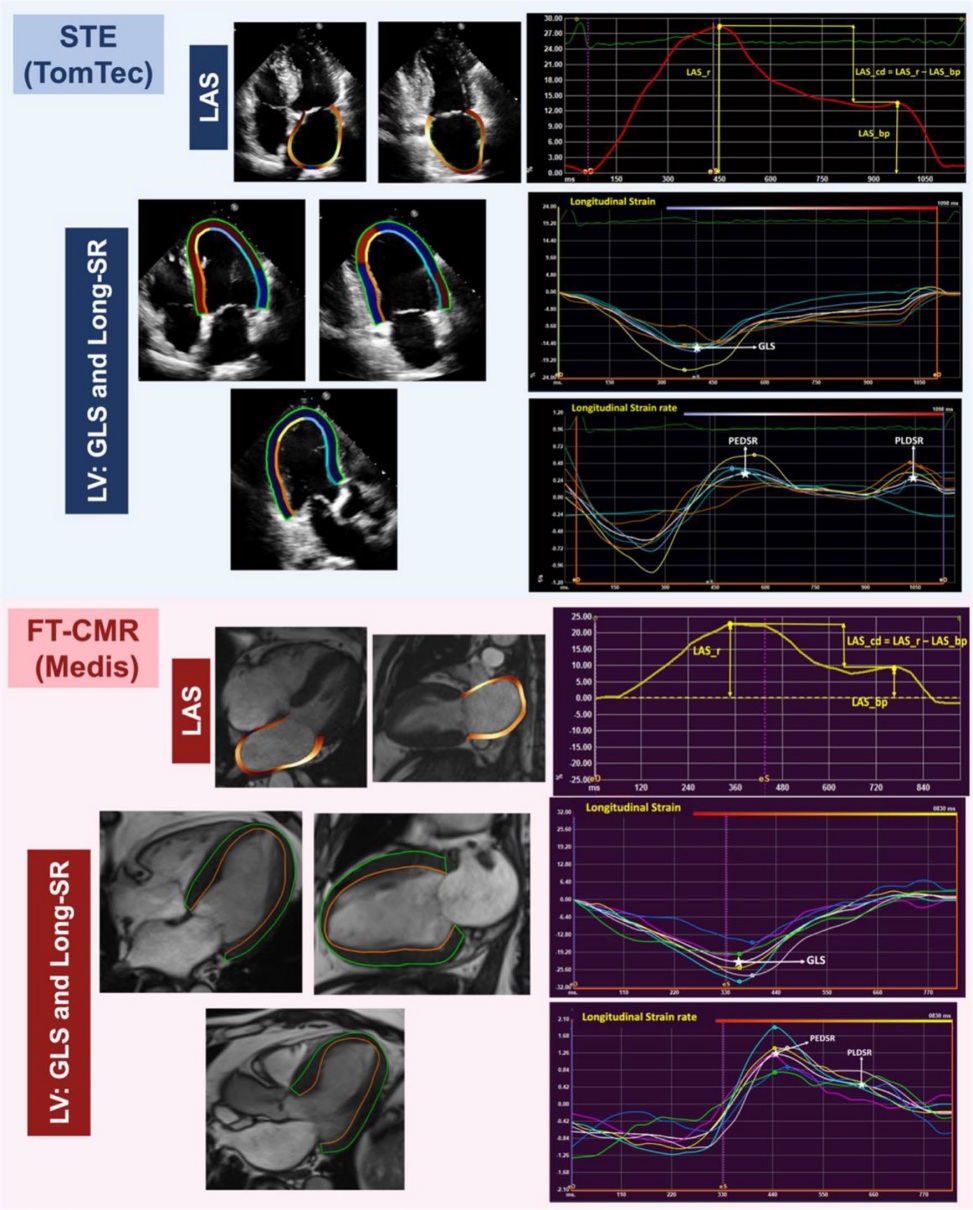
LA and LV longitudinal strain and LV diastolic strain rate analysis using Speckle Tracking Echocardiography (Upper) and Feature tracking-CMR (Lower). Abbreviations: STE = Speckle Tracking Echocardiography, LAS_r/cd/bp = Left atrial strain during reservoir/conduit/ booster pump phase, GLS = global longitudinal strain, SR = strain rate PE/PLDSR = peak early/peak late diastolic strain rate, FT-CMR = feature tracking– cardiovascular magnetic resonance imaging

Myocardial deformation (LA and LV) was assessed using TomTec-ARENA (v2.4, 2D-CPA) for STE and QStrain v2.0 (Medis v3.1, medical imaging system) for FT-CMR. Image analysis was conducted in the same manner on both STE and FT-CMR using semiautomated method (Fig. 1). LA endocardial borders and LV endo- and epicardial borders were traced at ventricular end-diastole and end-systole, excluding the LA appendage and pulmonary veins, then the software automatically propagated contours to the rest of the cardiac cycle. Contour adjustment was conducted if needed on the end-ventricular diastole and end-ventricular systole phases only. Strain and strain rate curves were obtained by identifying end ventricular diastole as the time reference (a value of zero designated as the baseline) [11]. LAS values corresponding to reservoir, conduit and booster pump function were obtained on 4- and 2-chamber long-axis views as illustrated in Fig. 1, with average values calculated. LV walls were traced on the three long-axis views for GLS and longitudinal peak early/ late diastolic strain rate (PE/PLDSR) (Fig. 1). LV mid circumferential strain (Mid-CS) and circumferential PE/PLDSR were calculated from the mid short axis view (Figure S1).

### Statistical analysis

Statistical analysis was performed using SPSS version 26.0 software. Normality was assessed using the Shapiro–Wilk test and histograms. Normally distributed data are expressed as mean ± standard deviation. Categorical data are expressed as counts and percentages. The differences between techniques or scans in the same cohort were assessed using paired t-test as the data were normally distributed. Both agreement and reproducibility were assessed using the Bland-Altman method [12] and two-way mixed-effect intraclass correlation coefficient (ICC) calculated for absolute agreement, with 95% confidence intervals (CI). For test-retest reproducibility, coefficient of variance (CoV) was also calculated to assess the range between the mean and standard deviation of the difference [13]. ICC was categorised as follows: excellent > 0.90, good 0.75–0.90, moderate 0.50–0.75 and poor < 0.50 [14], while CoV was scored as excellent < 10%, good 10–20%, moderate 20–30% and poor > 30%. All statistical tests were two-tailed, with a p-value < 0.05 considered statistically significant.

## Results

### Study population (Inter-technique agreement)

A total of 222 participants were included comprising: severe AS (*n* = 78), T2D (*n* = 87) and 57 healthy volunteers. Demographic data are presented in Table 1. Patients with severe AS were older and the majority were male, whilst those with T2D were heavier. Patients with severe AS (AV-Vmax = 4.32 ± 0.61 m/s, MPG = 45.2 ± 14.0mmHg and AVA = 0.84 ± 0.21cm^2^) demonstrated the highest CMR-measured indexed LA and LV volumes, LVMI and LV mass/volume ratio, and lowest.

EFs, compared to T2D and healthy volunteers.

**Table 1:** Baseline characteristics and CMR volumetric assessment of the study participants

Parameters	Healthy Volunteers (*n* = 57)	T2D (*n* = 87)	Severe AS (*n* = 78)
Age (years)	56.0 ± 12.1	50.5 ± 6.5	67.1 ± 9.4
Male (n (%))	34 (59)	51 (59)	58 (74)
Height (cm)	170.0 ± 9.0	168.0 ± 9.7	169.1 ± 9.7
Weight (kg)	74.0 ± 12.0	103.3 ± 16.7	84.8 ± 17.4
BMI (kg/m^2^)	25.0 ± 3.1	36.6 ± 5.5	29.6 ± 5.1
SBP (mmHg)	129.0 ± 19.2	139.6 ± 15.3	135.0 ± 21.1
DBP (mmHg)	78.0 ± 8.2	87.4 ± 8.0	75.9 ± 9.9
HR (bpm)	66.0 ± 10.6	74.4 ± 9.6	64.3 ± 15.9
Medical History			
Hypertension (n (%))	5 (8)	44 (51)	52 (66)
Hyperlipidaemia (n (%))	5 (8)	56 (64)	28 (35)
CMR Volumetric Assessment			
LVEDVi (ml/m^2^)	81.6 ± 16.1	61.8 ± 8.81	90.8 ± 17.6
LVESVi (ml/m^2^)	30.5 ± 8.0	21.6 ± 6.36	40.2 ± 11.0
LVEF (%)	62.8 ± 5.5	68.2 ± 6.72	56.0 ± 6.23
LVMi (g/m^2^)	53.0 ± 13.5	55.0 ± 9.0	74.7 ± 18.1
LVM/LVEDV (g/ml)	0.65 ± 0.11	0.82 ± 0.12	0.84 ± 0.23
LAVi_Max_ (ml/m^2^)	40.9 ± 9.5	31.3 ± 7.52	45.8 ± 14.8
LAVi_Min_ (ml/m^2^)	17.9 ± 5.0	13.7 ± 4.59	25.6 ± 12.3
LAEF (%)	56.4 ± 6.8	56.4 ± 7.87	45.5 ± 11.2

Data are n (%) or mean ± SD. Abbreviations: AF = atrial fibrillation, DBP = diastolic blood pressure, HR = heart rate, SBP = systolic blood pressure, LVED/ESVi = left ventricular end diastolic/end systolic volume indexed to body surface area, LVEF = left ventricular ejection fraction, LAVimax/min = Left atrial maximum/ minimum volume indexed to body surface area, LAEF = left atrial emptying fraction

**Fig. 2:**
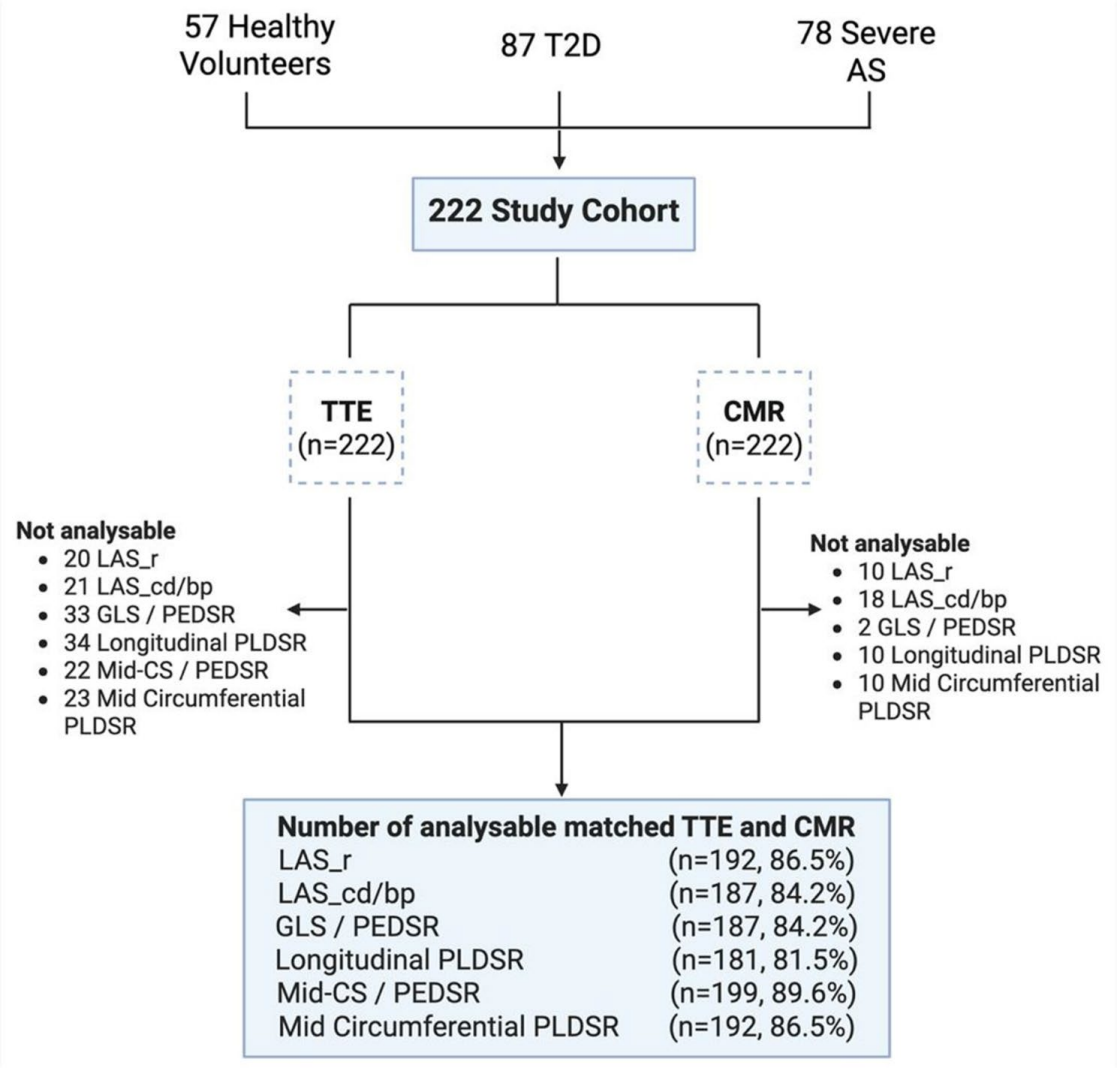
Number of analysable matched TTE and CMR images per parameter for inter-technique agreement assessment. The number of non-analysable images per parameter may include overlapping cases and does not necessarily represent unique patients. Abbreviations: T2D = Type two diabetes, AS = Aortic stenosis, LAS_r/cd/bp = Left atrial strain during reservoir/conduit/ booster pump phase, GLS/ PEDSR = global longitudinal strain and longitudinal peak early diastolic strain rate, Mid-CS/ PEDSR = Mid circumferential strain and Mid circumferential peak early diastolic strain rate, PLDSR = peak late diastolic strain rate

After pooling all cohorts and matching the analysable CMR and TTE scans for inter-technique agreement assessment (image quality ≥ 1), the numbers of subjects included for each parameter are listed in Fig. 2. Overall, 9–15% of the TTE and 0.9-8% of the CMR images were not analysable due to poor image quality or artefacts. Almost half of the unanalysable images on TTE (16/34) were from the T2D cohort due to body habitus, whilst for CMR the majority (13/18) were from the severe AS cohort where prospective ECG-gating was used due to ectopics or atrial fibrillation.

### Inter-technique agreement

The results for the inter-technique agreement of LA and LV deformation parameters are shown in Table 2.

**Table 2:** Inter-technique agreement of LA and LV parameters CMR vs. TTE

Parameter	CMR (Mean ± SD)	TTE (Mean ± SD)	*p*-value	Bias (Limits of agreement)	ICC	95% CI
**Left Atrial**						
LAS_r (%)	30.3 ± 8.79	31.2 ± 7.51	0.145	-0.89 (-17.45, 15.7)	0.63	0.51−0.72
LAS_cd (%)	15.6 ± 6.84	16.1 ± 6.45	0.321	-0.47(-13.1, 12.2)	0.69	0.59−0.77
LAS_bp (%)	15.0 ± 5.14	15.3 ± 5.0	0.458	-0.31 (-11.4, 10.7)	0.55	0.40−0.67
**Left Ventricular**						
GLS (%)	-17.2 ± 3.31	-15.8 ± 2.90	< 0.001*	-1.39(-8.31, 5.53)	0.49	0.30−0.63
Longitudinal PEDSR (s^− 1^)	0.68 ± 0.20	0.48 ± 0.14	< 0.001*	0.20 (-0.16, 0.56)	0.44	-0.13−0.70
Longitudinal PLDSR (s^− 1^)	0.44 ± 0.16	0.37 ± 0.11	< 0.001*	0.07 (-0.18, 0.33)	0.66	0.41−0.80
Mid-CS (%)	-19.3 ± 3.19	-23.0 ± 4.37	< 0.001*	3.63 (-5.77,13.0)	0.26	-0.03−0.46
Mid Circumferential PEDSR (s^− 1^)	0.96 ± 0.33	0.88 ± 0.24	0.002*	0.08 (-0.63, 0.80)	0.33	0.12−0.49
Mid Circumferential PLDSR (s^− 1^)	0.48 ± 0.25	0.47 ± 0.22	0.595	0.01 (-0.51, 0.53)	0.52	0.37−0.64

Data are n (%), mean ± SD. Asterisks (*) and bold values indicate statistically significant differences (P < 0.05)

Abbreviations: LAS_r/cd/bp = Left atrial strain during reservoir/conduit/ booster pump phase, GLS = global longitudinal strain, PE/PLDSR = peak early/peak late diastolic strain rate, Mid_CS = Mid circumferential strain

### LA strain

There was no significant difference in LAS values (LAS_r, LAS_cd or LAS_bp) between CMR and TTE. There was moderate inter-technique agreement for all LAS parameters between CMR and TTE (ICC = 0.55–0.69).

### LV strain

For LV parameters, most strain and SR values were significantly higher on CMR than TTE, apart from Mid circumferential PLDSR which was no different, and Mid-CS which was significantly higher on TTE. The inter-technique agreement between CMR and TTE was moderate for longitudinal and circumferential PLDSR (ICC 0.66 and 0.52 respectively), but poor for all other parameters. GLS showed better agreement between the two modalities than Mid-CS.

Inter-technique agreement in the different disease states is shown in Supplemental Table S1. Overall, the results were similar to the pooled analysis. However, agreement between STE and FT-CMR parameters was significantly lower in the T2D cohort compared to healthy volunteers and AS cohorts.

### Test-retest reproducibility

The mean age of the test-retest cohort with T2D (*n* = 10) was 65.6 ± 7.3 years and 50% were male. The mean interval between the two study visits was 11 ± 4 days, with no significant difference in the hemodynamic measurements between the two scans (Table S2). All TTE and CMR images were analysable (*n* = 20 each). CMR image quality was rated as: excellent (*n* = 12, 60%) or good (*n* = 8, 40%), while TTE image quality was: good (*n* = 17, 85%) or suboptimal (*n* = 3, 15%).

**Fig. 3:**
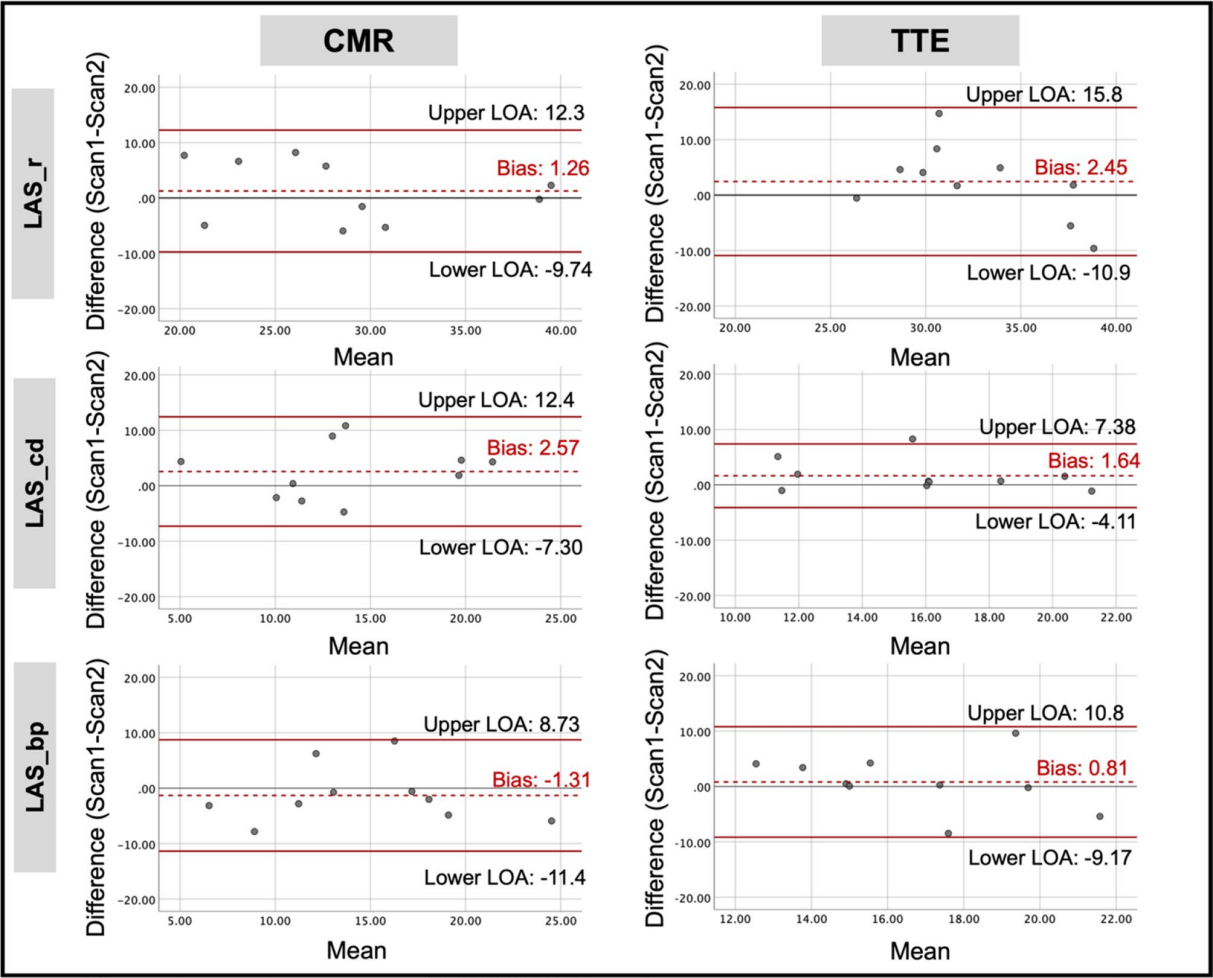
Bland-Altman plots for test-retest reproducibility of LA strain parameters using CMR and TTE. Abbreviations: LAS_r/cd/bp = Left atrial strain during reservoir/conduit/ booster pump phases, LOA = Limits of agreement

The test-retest reproducibility of LA and LV parameters by CMR and TTE are shown in Table 3. There was no significant difference in any LA or LV deformation parameter between scan-1 and scan-2 on either modality. For LA parameters, the test-retest reproducibility was good for reservoir and booster pump-LAS on CMR, whilst conduit-LAS had good test-retest reproducibility on TTE (Fig. 3).

**Fig. 4:**
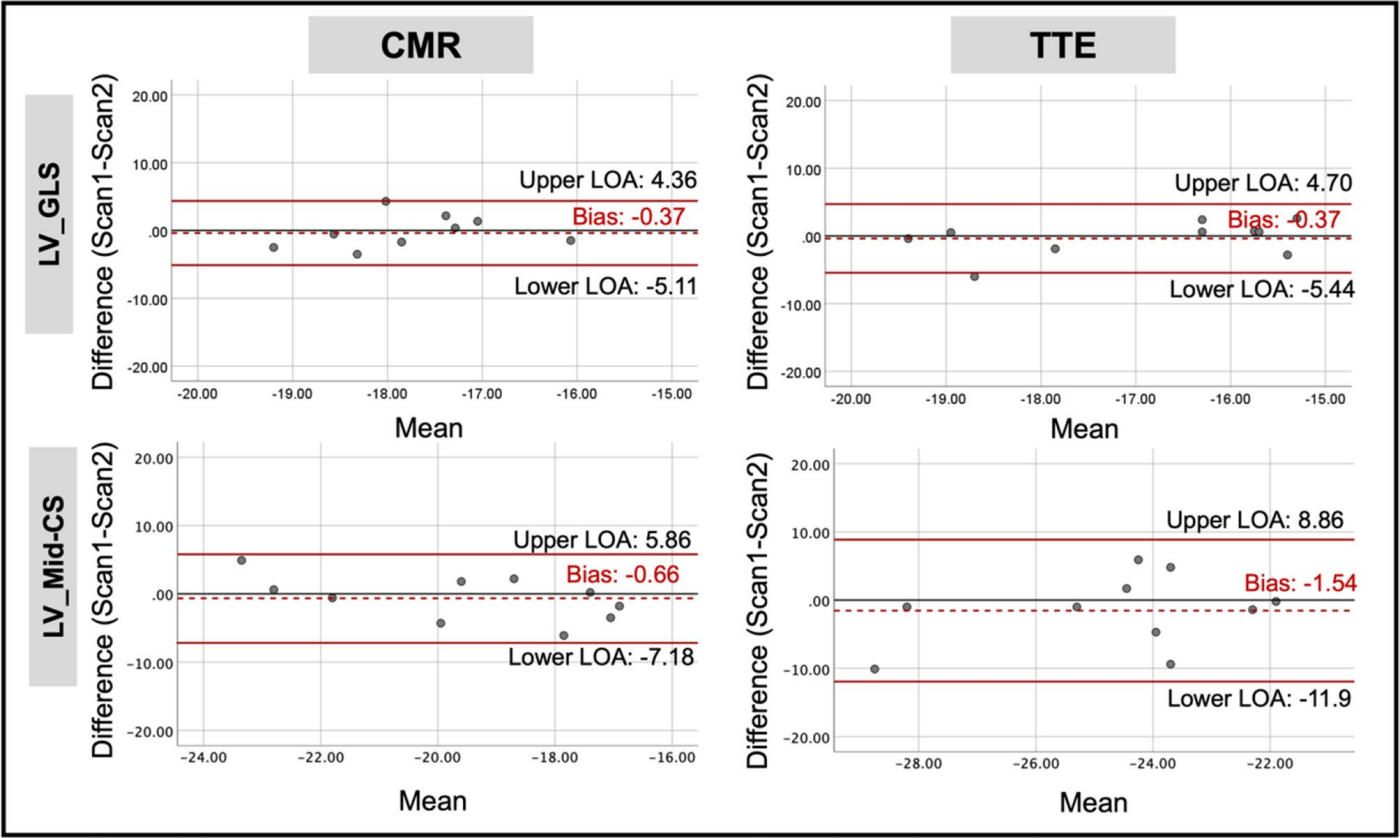
Bland-Altman plots for test-retest reproducibility of LV global longitudinal and mid-circumferential strain using CMR and TTE. Abbreviations: LV_GLS = left ventricular global longitudinal strain, Mid-CS = Mid circumferential strain

For the LV, GLS had almost identical bias and limits of agreement on Bland-Altman analysis and similar CoV (~ 15%) on both imaging modalities, but higher ICC (0.65 vs. 0.36) on CMR compared to TTE. Mid-CS demonstrated better test-retest reproducibility on CMR, with narrower limits of agreement and higher ICC compared to TTE (Fig. 4). Both CMR and TTE showed good test-retest reproducibility for most PE/PLDSR parameters.

**Table 3:** Test-retest reproducibility of LA and LV parameters using CMR vs. TTE

Parameters	CMR					TTE				
	Scan 1 (Mean ± SD)	Scan 2 (Mean ± SD)	Bias (Limits of agreement)	ICC	CoV (%)	Scan 1 (Mean ± SD)	Scan 2 (Mean ± SD)	Bias (Limits of agreement)	ICC	CoV (%)
LAS_r (%)	29.2 ± 6.5	27.9 ± 7.8	1.26 (-9.74, 2.3)	0.83	19.6	33.8 ± 3.70	31.4 ± 6.76	2.45 (-10.9, 15.8)	0.35	20.9
LAS_cd (%)	15.1 ± 6.0	12.6 ± 5.3	2.57 (-7.30, 12.4)	0.73	36.3	16.7 ± 3.41	15.0 ± 4.16	1.64 (-4.11, 7.38)	0.80	18.5
LAS_bp (%)	14.0 ± 5.9	15.4 ± 6.0	-1.31 (-11.4, 8.73)	0.78	34.9	17.1 ± 3.18	16.3 ± 4.41	0.81 (-9.17, 10.8)	0.23	30.4
GLS (%)	-17.4 ± 2.2	-17.0 ± 2.5	-0.37 (-5.11,4.36)	0.65	14.1	-17.2 ± 2.4	-16.8 ± 1.6	-0.37 (-5.44, 4.70)	0.36	15.3
Longitudinal PEDSR (s^− 1^)	0.73 ± 0.24	0.66 ± 0.22	0.08 (-0.22,0.37)	0.86	21.8	0.61 ± 0.12	0.60 ± 0.12	0.01 (-0.20, 0.22)	0.79	17.3
Longitudinal PLDSR (s^− 1^)	0.53 ± 0.17	0.54 ± 0.25	-0.01 (-0.29,0.27)	0.88	26.7	0.48 ± 0.04	0.46 ± 0.05	0.02 (-0.12, 0.16)	0.35	15.1
Mid_CS (%)	-19.9 ± 2.0	-19.2 ± 3.6	-0.66 (-7.18,5.86)	0.53	17.0	-25.4 ± 4.0	-23.9 ± 2.8	-1.54 (-11.9, 8.86)	0.40	21.5
Mid Circumferential PEDSR (s^− 1^)	1.02 ± 0.32	0.95 ± 0.33	0.08 (-0.30,0.45)	0.90	19.6	0.88 ± 0.26	0.79 ± 0.18	0.09 (-0.29, 0.46)	0.75	23.1
Mid Circumferential PLDSR (s^− 1^)	0.64 ± 0.32	0.67 ± 0.23	-0.03 (-0.44,0.38)	0.85	32.0	0.68 ± 0.20	0.63 ± 0.11	0.05 (-0.21, 0.30)	0.80	20.2

Abbreviations as Table 2

## Discussion

This is the first study to investigate the agreement between TTE and CMR for measuring LA and LV myocardial deformation assessment in a large cohort of participants with a range of diseases, with prospectively acquired images at the same research visit. Moreover, this is also the first study to assess the test-retest reproducibility of both TTE and CMR-derived LA and LV myocardial deformation parameters in the same cohort, albeit in a small number. Our results showed that TTE image analysis was more limited by image quality, with up to 15% of the images not analysable. The overall inter-technique agreement was moderate for LAS and moderate to poor for LVS/DSR, suggesting these techniques cannot be used interchangeably. We found CMR showed better test-retest reproducibility for reservoir and booster pump LAS, whilst TTE showed better reproducibility for conduit LAS. The test-retest reproducibility for LV GLS was good on CMR and poor on TTE.

### Inter-technique agreement

This study showed moderate inter-technique agreement for LA and LV myocardial deformation assessment. We used QStrain from Medis for the CMR image analysis and Tomtec for the TTE images. Both Medis and Tomtec use optical flow technology to calculate strain and SR, which has been provided by Advanced Medical Imaging Development (AMID) software [15, 16]. This should therefore minimise variability related to image analysis software vendors [17, 18].

There are limited publications on the inter-technique agreement of myocardial deformation parameters. One LA-focused study included patients (*n* = 43) and healthy volunteers (*n* = 11) who underwent CMR and TTE scans on the same day and images were analysed twice using two different software packages for each imaging modality [19]. This showed good inter-technique agreement for all three LAS parameters with systematic differences between vendors in both modalities [19]. On the contrary, a recent retrospective study, which included 70 patients with a range of cardiac diseases, showed reservoir and conduit strain by CMR to be systematically higher than TTE, with significant inter-technique proportional bias [20]. This is likely to be due to the time difference between the CMR and TTE scans in this study (within 6 months of each other), allowing the change in loading conditions of the atria to influence the results.

Only one modest sized study focused on LV deformation, which included 39 patients with various cardiac diseases and 41 controls, and showed LV-GLS had better inter-technique agreement than GCS, when the TTE and CMR scans were performed on the same day (bias ± limits of agreement = 0.21 ± 3.68% vs. − 1.33 ± 6.86%, respectively) [21]. This was despite using different image analysis software developers (EchoPAC by GE for STE and Circle software for CMR-FT), suggesting the inter-changeable use of both imaging modalities and different vendors for monitoring GLS. However, our results do not support this, as the agreement between the two imaging techniques was modest to poor. This could be attributed to our larger and more heterogeneous cohort which is important to obtain a wide range of values as seen in practice. The variability could also be related to the difference in the contrast-to-noise ratio between 1.5T and 3T MRI scanners. In the afore-mentioned study, CMR scans were conducted using 1.5T [21], while in our study both 1.5T and 3T MRI scanners were used, which could impact the strain/SR values by FT-CMR [22].

Our large cohort allowed us to explore the inter-technique agreement in healthy volunteers versus patients with T2D or AS. The overall agreement between STE and FT-CMR parameters was significantly lower in the T2D cohort, likely due to the T2D cohort being overweight, which limited TTE image quality.

### Test-retest reproducibility

Only one previous study assessed test-retest reproducibility of LA function using CMR alone in 22 healthy volunteers [23]. This showed modest test-retest reproducibility for reservoir strain (ICC = 0.60), whilst our results demonstrated higher reproducibility (ICC = 0.83). The reason for better reservoir and booster-pump LAS reproducibility by CMR compared to TTE could be attributed to the higher spatial resolution of CMR, with high contrast ratio between the blood pool and myocardium. Moreover, TTE scanning is operator-dependent and prone to foreshortening of the LA, which limits tracking LA maximum and minimum lengths (reservoir and booster-pump). Although the ICCs in our study were high for LAS values, indicating good consistency between repeated measures, the CoVs were also relatively high for conduit and booster-pump LAS, suggesting notable variability in absolute values. This level of variability could limit the clinical utility of LAS measurements, especially when precise cut-offs are needed for decision-making.

This study showed that GLS had very similar reproducibility on both CMR and TTE, whilst CMR had slightly better reproducibility for circumferential strain. There is only one recent study comparing the test-retest reproducibility of LVS by CMR and TTE, which included 20 patients with heart failure and 30 controls [24]. The study reported that GLS by STE had lower variability than GLS by CMR-FT (CoV 8.9 vs. 15.1%). However, the Bland-Altman analysis in that study showed a smaller bias for FT than STE (Bias 0.08 ± 2.22 vs. -0.21 ± 2.14, respectively). The software packages used were by different developers, TomTec for STE and Cvi42 for FT, in that study, which could have added to the difference in the CoV, as each software has a different algorithm to calculate strain and the reproducibility of the software varies [18, 19].

Other TTE studies assessing GLS test-retest reproducibility demonstrated higher ICC values than our results (ICC > 0.90) [28, 29]. The discrepancy could be attributed to the difference in the studied population: our study included people with T2D and a high BMI (> 30 kg/m2), which limited the TTE acoustic window.

### Clinical implications

The moderate to poor agreement between CMR and TTE for assessing LA and LV myocardial deformation shown in our study suggests that these techniques should not be used interchangeably in clinical settings. This is particularly relevant given that guideline-based clinical decisions, such as the use of LV-GLS and LA reservoir strain for risk stratification or treatment guidance, rely on precise and consistent measurements. Moreover, the suggested cut-off values for strain in the published guidelines are TTE generated, and should therefore be used with caution when using CMR for its assessment. Further population-based studies are required to generate and validate LA and LV myocardial deformation parameters taking into account gender, ethnicity, MRI strength, vendor and inter-technique differences [25].

### Study strengths and limitations

This study has several strengths. It is the largest study to date with prospectively recruited research participants who underwent same-day TTE and CMR. We conducted rigorous blinding and assessment of image quality, using the same analysis method by a single observer. The study included healthy volunteers and participants with evidence of subclinical cardiac dysfunction, allowing a wider overview that is more clinically applicable. It is the first study to extensively assess LAS by TTE and CMR in a large, heterogeneous cohort, and the only study to assess the test-retest reproducibility of both TTE and CMR-assessed LA and LV deformation parameters in the same cohort.

We also acknowledge several limitations. The study included data from CMR scans conducted using 1.5T and 3T scanners, as well as from two different TTE vendors. Moreover, the test-retest scans on both modalities were performed by different radiographers/physiologists. However, this reflects standard clinical practice. The generalizability of test-retest results is limited by the small subgroup size, which included T2D only. However, 10 subjects allow 80% power for an ICC of 0.7, as published previously [26]. Intra- and inter-observer variability analyses were not included in this study. However, test-retest assessment incorporates intra-observer variability as the analysis was done by the same observer. Moreover, previous publications have shown good to excellent intra- and inter-observer variability in larger cohorts for LV [21, 27, 28] and LA [19, 29, 30] assessment. Inter-vendor differences between analysis software are not known and further studies are needed. The study did not explore the association between LA and LV deformation parameters and clinical outcomes, as its primary aim was to assess the inter-technique agreement between TTE and CMR measurements. Future prospective studies incorporating longitudinal outcomes and therapeutic decision-making are essential to determine which modality provides more clinically actionable or prognostically relevant data in specific populations such as AS or T2D. Given the exploratory nature of this study, no formal correction for multiple comparisons was applied. This may increase the likelihood of type I error, and thus, findings should be interpreted with caution and validated in larger studies.

## Conclusions

In subjects with or without cardiovascular disease, the inter-technique agreement between CMR and TTE was modest for LAS parameters, whilst the agreement was moderate to poor for LV myocardial deformation, suggesting the two imaging modalities cannot be used interchangeably. Despite the lack of interchangeability, both LV and LA strain remain integral to contemporary cardiology practice, offering critical insights into myocardial and atrial function. In people with T2D, CMR showed better test-retest reproducibility for reservoir and booster pump-LAS and Mid-LV circumferential strain, whilst the test-retest reproducibility of GLS and most LVDSR parameters were similar on CMR and TTE.

## Electronic supplementary material

Below is the link to the electronic supplementary material.

Supplementary Material 1

## Data Availability

The datasets used and/or analysed during the current study are available from the corresponding author on reasonable request.

## Abbreviations

AS: Aortic stenosis
GLS: Global longitudinal strain
LA: Left atrium
LAS: bp– Left atrial strain during booster pump phase
LAS_cd: Left atrial strain during conduit phase
LAS_r: Left atrial strain during reservoir phase
Mid-CS: Mid circumferential strain
PE/PLDSR: Peak early/peak late diastolic strain rate
T2D: Type 2 diabetes
