# Supplementary material for: Inter-technique agreement of left atrial and ventricular deformation analysis: a comparison between transthoracic echocardiography and cardiovascular magnetic resonance imaging
Source: Echo Res Pract. 2025 Oct 6;12:24. doi: 10.1186/s44156-025-00090-3 (PMC12498453; doi:10.1186/s44156-025-00090-3)
Supplement: Supplementary file 1 — Supplementary Material 1 [file 44156_2025_90_MOESM1_ESM.docx]

# Online Supplementary Data

**Table S1:** **Inter-technique agreement of LA and LV deformation parameters across diseases states CMR vs TTE.**

| Parameter | CMR  (Mean±SD) | TTE  (Mean±SD) | p-value | Bias  (Limits of agreement) | ICC | 95% CI |
| --- | --- | --- | --- | --- | --- | --- |
| Severe AS (n=78) | | | | | |  |
| LAS_r (%) | 24.4 ± 7.3 | 24.7 ± 5.7 | 0.78 | -0.23 (-13.2, 12.7) | 0.66 | 0.44−0.79 |
| LAS_cd (%) | 11.2 ± 4.6 | 10.3 ± 4.2 | 0.21 | 0.86 (-9.5, 11.2) | 0.44 | 0.07−0.66 |
| LAS_bp (%) | 13.9 ± 5.0 | 14.6 ± 4.7 | 0.29 | -0.67 (-10.1, 8.8) | 0.68 | 0.47−0.81 |
| GLS (%) | -14.9 ± 3.0 | -14.0 ± 2.5 | **0.04*** | -0.87 (-7.38, 5.64) | 0.42 | 0.06−0.65 |
| Long_PEDSR (s^-1^) | 0.58 ± 0.18 | 0.42 ± 0.11 | **<0.001*** | 0.18 (-0.16, 0.52) | 0.32 | -0.15−0.60 |
| Long_PLDSR (s^-1^) | 0.49 ± 0.20 | 0.39 ± 0.11 | **<0.001*** | 0.09 (-0.19, 0.37) | 0.69 | 0.34−0.84 |
| Mid-CS (%) | -19.7 ± 3.0 | -23.6 ± 4.7 | **<0.001*** | 3.94 (-5.79, 13.7) | 0.26 | -0.12−0.52 |
| Mid-Circ_PEDSR (s^-1^) | 0.84 ± 0.36 | 0.82 ± 0.23 | 0.77 | 0.01 (-0.77, 0.80) | 0.22 | -0.28−0.52 |
| Mid-Circ_PLDSR (s^-1^) | 0.58 ± 0.29 | 0.50 ± 0.22 | **0.05*** | 0.07 (-0.46, 0.61) | 0.61 | 0.36−0.77 |
| T2D (n=87) | | | | | |  |
| LAS_r (%) | 31.6 ± 7.2 | 34.4 ± 6.3 | **0.01*** | -2.87 (-21.4, 15.7) | 0.04 | -0.46−0.38 |
| LAS_cd (%) | 16.1 ± 5.4 | 18.1 ± 4.6 | **0.009*** | -1.97 (-14.3, 10.4) | 0.32 | -0.05−0.56 |
| LAS_bp (%) | 15.5 ± 4.8 | 16.4 ± 4.6 | 0.147 | -0.91 (-11.3, 9.5) | 0.53 | 0.25−0.70 |
| GLS (%) | -17.9 ± 3.2 | -15.2 ± 2.4 | **<0.001*** | -2.68 (-10.3, 4.9) | 0.08 | -0.26−0.36 |
| Long_PEDSR (s^-1^) | 0.68 ± 0.2 | 0.42 ± 0.1 | **<0.001*** | 0.25 (-0.10, 0.61) | 0.13 | -0.14−0.38 |
| Long_PLDSR (s^-1^) | 0.37 ± 0.1 | 0.31 ± 0.1 | **<0.001*** | 0.06 (-0.15, 0.28) | 0.29 | -0.08−0.55 |
| Mid-CS (%) | -19.5 ± 3.3 | -22.4 ± 4.0 | **<0.001*** | 2.83 (-6.56, 12.3) | 0.21 | -0.13−0.47 |
| Mid-Circ_PEDSR (s^-1^) | 1.10 ± 0.3 | 0.91 ± 0.2 | **<0.001*** | 0.19 (-0.46, 0.84) | 0.31 | -0.04−0.55 |
| Mid-Circ_PLDSR (s^-1^) | 0.45 ± 0.19 | 0.51 ± 0.23 | **0.05*** | -0.06 (-0.61, 0.48) | 0.21 | -0.21−0.49 |
| Healthy Volunteers (n=57) | | | | | |  |
| LAS_r (%) | 35.6 ± 8.3 | 34.6 ± 5.6 | 0.37 | 1.06 (-15.7, 17.8) | 0.43 | 0.02−0.67 |
| LAS_cd (%) | 20.0 ± 7.7 | 19.9 ± 6.3 | 0.91 | 0.12 (-14.5, 14.7) | 0.62 | 0.34−0.78 |
| LAS_bp (%) | 15.6 ± 5.6 | 14.6 ± 5.6 | 0.31 | 0.94 (-12.3, 14.1) | 0.43 | 0.02−0.67 |
| GLS (%) | -18.8 ± 2.3 | -18.4 ± 1.8 | 0.33 | -0.37 (-5.89, 5.14) | 0.17 | -0.42−0.51 |
| Long_PEDSR (s^-1^) | 0.79 ± 0.21 | 0.63 ± 0.14 | **<0.001*** | 0.16 (-0.20, 0.53) | 0.47 | -0.06−0.72 |
| Long_PLDSR (s^-1^) | 0.48 ± 0.18 | 0.41 ± 0.09 | **<0.001*** | 0.07 (-0.20, 0.34) | 0.64 | 0.34−0.80 |
| Mid-CS (%) | -18.6 ± 3.1 | -23.1 ± 4.3 | **<0.001*** | 4.44 (-4.32, 13.2) | 0.30 | -0.15−0.59 |
| Mid-Circ_PEDSR (s^-1^) | 0.91 ± 0.26 | 0.90 ± 0.24 | 0.81 | 0.01 (-0.62, 0.64) | 0.32 | -0.19−0.61 |
| Mid-Circ_PLDSR (s^-1^) | 0.42 ± 0.23 | 0.37 ± 0.17 | 0.09 | 0.05 (-0.36, 0.47) | 0.62 | 0.36−0.78 |

Abbreviations: LAS_r/cd/bp = Left atrial strain at reservoir/conduit/ booster pump phase, GLS= global longitudinal strain, Long_PE/PLDSR= longitudinal peak early/peak late diastolic strain rate, Mid-CS= Mid circumferential strain. Mid-Circ_PE/PLDSR= Mid circumferential peak early/peak late diastolic strain rate.

**Table S2: Hemodynamic measurements for test-retest reproducibility**

|  | Scan 1 | Scan 2 | p-value |
| --- | --- | --- | --- |
| CMR | | | |
| SBP (mmHg) | 136.0 ± 228 | 135.8 ± 24.5 | 0.965 |
| DBP (mmHg) | 82.4 ± 9.5 | 79.5 ± 11.3 | 0.361 |
| HR (bpm) | 76.5 ± 11.7 | 73.1 ± 13.0 | 0.236 |
| TTE | | | |
| SBP (mmHg) | 130.8 ± 16.5 | 131.5 ± 16.5 | 0.235 |
| DBP (mmHg) | 83.7 ± 4.2 | 82.7 ± 1.9 | 0.363 |
| HR (bpm) | 75.8 ± 8.5 | 73.9 ± 10.2 | 0.305 |

Abbreviations: DBP= diastolic blood pressure, HR= heart rate, SBP= systolic blood pressure.


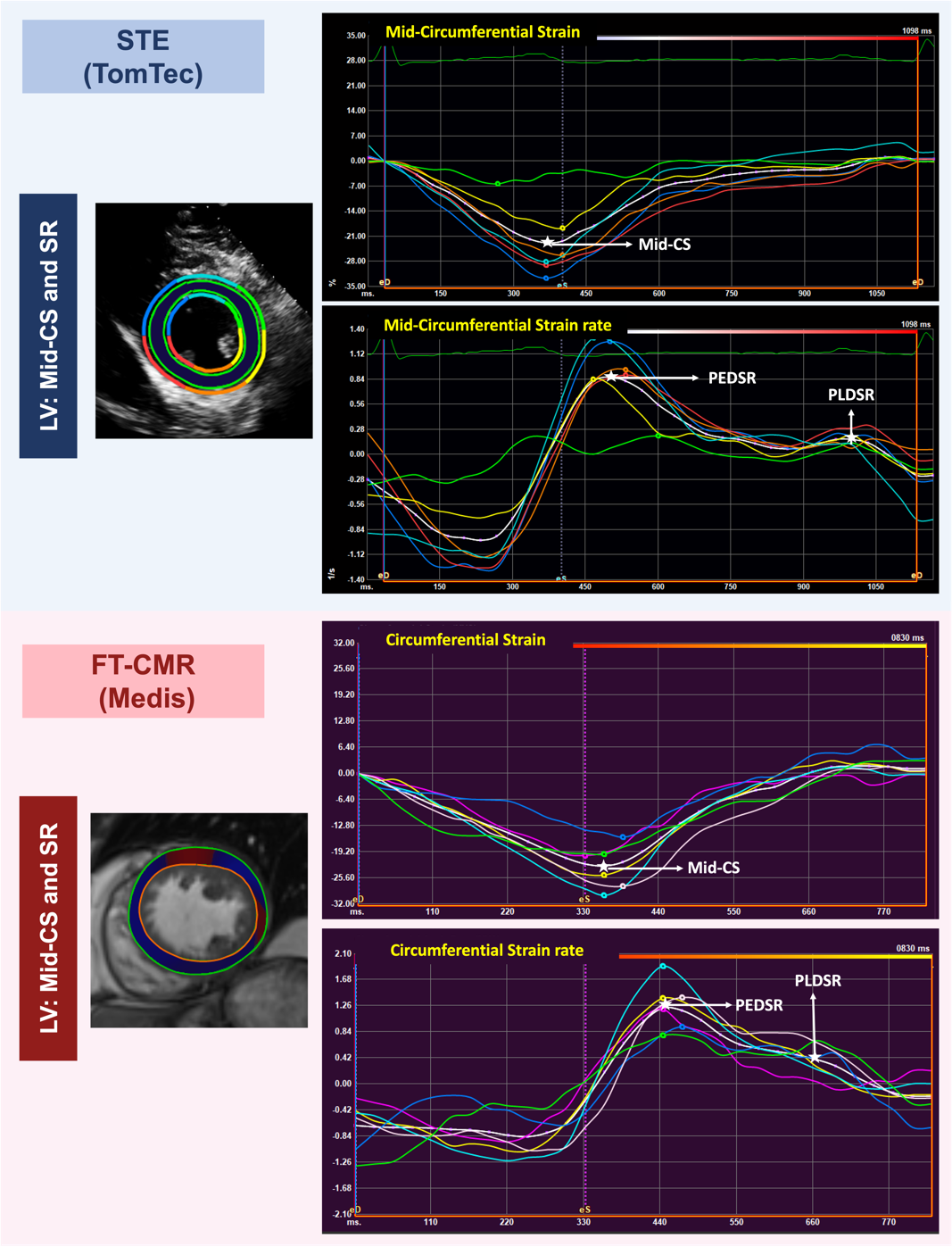


**Figure S1: LV mid circumferential strain and diastolic strain rate analysis using Speckle Tracking Echocardiography (Upper) and Feature tracking-CMR (Lower).**

Abbreviations: Mid-CS= Mid circumferential strain. PE/PLDSR= peak early/peak late diastolic strain rate.
